# Optimization of Process Parameters for Anti-Glare Spray Coating by Pressure-Feed Type Automatic Air Spray Gun Using Response Surface Methodology

**DOI:** 10.3390/ma12050751

**Published:** 2019-03-05

**Authors:** Yu-Hui Huang, Lung-Chuan Chen, Huann-Ming Chou

**Affiliations:** 1 Graduate School of Mechanical and Energy Engineering, Kun Shan University, 71070 Tainan, Taiwan; 2 Department of Materials Engineering, Kun Shan University, 71070 Tainan, Taiwan; 3 Department of Mechanical and Energy Engineering, Kun Shan University, 71070 Tainan, Taiwan; hmchou@mail.ksu.edu.tw

**Keywords:** anti-glare, spray-coating, gloss, haze, response surface methodology

## Abstract

The process of preparing anti-glare thin films by spray-coating silica sol-gel to soda-lime glass was exclusively and statistically studied in this paper. The effects of sol-gel deliver pressure, air transport pressure, and spray gun displacement speed on the gloss, haze, arithmetic mean surface roughness, and total transmittance light were analyzed. The experimental results indicate that the factors of sol-gel deliver pressure, air transport pressure, and displacement speed exhibit a significant effect on the haze, gloss, and Ra. In contrast, the variation of total transmittance light with these three factors are insignificant. Because the anti-glare property is predominantly determined by low gloss and high haze, we therefore aim to minimize gloss and maximize haze to achieve high anti-glare. Central composite design and response surface methodology are employed to analyze the main and interaction effects of the three factors through quadratic polynomial equations, which are confirmed by the analysis of variance and R^2^. The response surface methodology predict the lowest gloss and highest haze are 9.2 GU and 57.0%, corresponding to the sol-gel deliver pressure, air-transport pressure, and displacement speed of 250 kPa, 560 kPa, and 140 mm/s, and 340 kPa, 620 kPa, and 20 mm/s, respectively. Comparing the predicted optimal data with the real experimental results validates the applicability of the mathematical model. This study provides an important basis for the subsequent production of anti-glare glass with different specifications to satisfy the market demand.

## 1. Introduction

**Figure 1:**
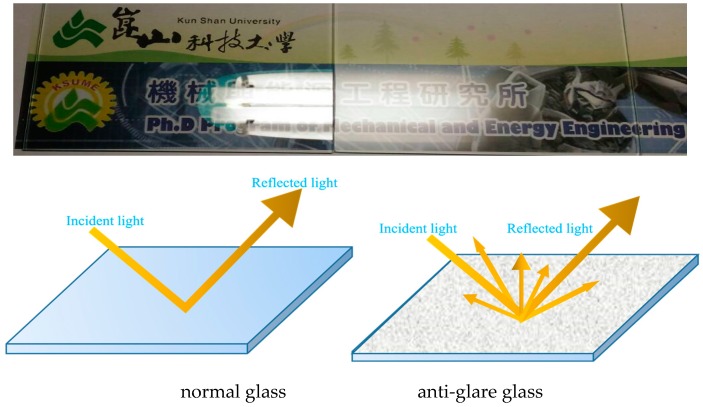
Schematic diagram of light path for normal and anti-glare glass.

Mobile phones have become a necessity for life and attracted more and more attention. In terms of increasing portability, the mobile phones are requested to be lighter, thinner, and better texture. In addition, the requirement of mobile phones for entertainment is increasing day by day. Consequentially, the demand for visual and operational convenience is also relatively increasing, which in turn drives the smartphone technology to be constantly updated and makes the application field more extensive. Anti-glare treatment could reduce the high light intensity and glare caused by excessive concentration of light, thereby improving the user’s comfort for a cover lens. The mechanism of anti-glare is depicted in Figure 1. 

At present, surface roughening is the predominant route to prepare anti-glare thin films, and the related methods involve particle blast [1], solution etching [2,3,4], solution spin/dip coating [5,6,7], solution spraying [8,9,10,11,12,13], and imprint [14,15,16,17,18,19,20]. In general, sand, fine metal, and ceramic particles are used to blast the substrate to roughen the surface and create a granulation-like depression. Chen [1] applied glass beads to blast acrylic materials to obtain round holes and atomized surfaces. Anti-glare glass can also be prepared by etching the substrates using chemical solution, such as hydrofluoric acid [2,3,4]. However, this method is not environmentally friendly and is hazardous for health. Haga et al. [5] disclosed an anti-glare film having fine irregularities with an averaged surface roughness of 0.05 to 0.5 μm by spin coating or dip coating using a resin solution containing specific additive particles. Liu et al. [6] fabricated light-scattering particles (LSPs), which were mainly constituted by polystyrene microbeads with amino groups and aliphatic chains, in order to evaluate the effects of the surface functional groups and the nature of the resins on the haze of anti-glare (AG) films. They suggested that the outer haze was mainly affected by the interaction between LSPs and the resins. Cho [7] demonstrated the antireflection coating of SiO_2_ nanospheres for cover glass by using a spin-coating method. However, this process suffered from an uneven coating of particles and lowered the transmittance of the substrate. Schmidt [8] et al. used a hydrolysis-condensation method and raw materials of tetraethoxysilane (TEOS) and methyl triethoxysilane (MTES) to prepare a sol-gel solution, which was then added with water-soluble tin oxide and sprayed onto a glass substrate. The materials were cured at 500 °C to roughen the surface to form an anti-glare glass. Aegerter et al. [9,10] sprayed (3-Glycidyloxypropyl) trimethoxysilane (GPTMS) or 3-(Mercaptopropyl) trimethoxysilane (MPTMS) sol-gel solution with an additive of indium tin oxide to plastic substrates of polymethyl methacrylate and polycarbonate to form an anti-glare and anti-static plastic substrate after curing at 130 °C under irradiation of ultra-violet. However, the substrate’s surface was too rough owing to the presence of metallic compounds. Yeh [11] sprayed a resin-based sol-gel solution on a pre-treated hard coating PMMA sheet to form an anti-glare layer. Additionally, Tri Rakhmawati [12] sprayed spherical silica powder onto a glass substrate to act as a light scattering layer. However, this powder tended to aggregate with each other due to high surface energy, causing poor dispersion efficiency. Ma et al. [13] modified the surface of silica nano-particles with cationic surfactant cetyltrimethylammonium bromide (CTAB) to improve surface aggregation phenomenon. Whitesides et al. [14,15] proposed a micro-contact transfer film process using the self-assembly monolayer to transfer the pattern onto the mold to the substrate. 

Chou et al. [16] proposed nanoimprint lithography by using the method of changing temperature and pressure to develop “hot embossing”. However, the hot-pressed nano-transfer process possessed a great disadvantage that the mold had a thermal expansion problem in the state of high temperature and high pressure, which caused a dimensional error in subsequent pattern transfer. The thermal deformation of the structure was more serious under a higher pressure. Later, Bailey et al. [17] improved the procedures of hot pressing to retard thermal deformation, but it was not appropriate for a large area. 

Although coating techniques and sol-gel synthesis have advanced considerably [18,19], there are still some technical challenges in the uniformity of nanoparticle distribution on substrates. It has been over 30 years since the introduction of a spray-coating process with the sol-gel particles; however, until now, the optimization conditions of these processes to produce anti-glare thin films are still far from clear because they are affected by many variables and their interactions [20]. Therefore, appropriate instruments and their optimization are essential to improve the performance of the spraying system with silica sol-gel solution without additives. Hence, this study will adopt central composite design and response surface methodology (RSM) to explore the regulation of anti-glare sol-gel spray parameters and their optimization. An empirical regression equation is also obtained to describe the quantitative effects of the operating parameters on the anti-glare.

## 2. Materials and Methods

### 2.1. Materials

High purity tetraethoxysilane (TEOS) and methyltrimethoxysilane (MTMS) were purchased from Evonik (Evonik, Hanau-Wolfgang, Germany). Nitric acid (EP) was purchased from Union Chemical Works (Union Chemical Works Ltd., Hsin-Chu, Taiwan). Methanol were all of 99.9% purity and obtained from Shiny Chemical Industry Co. (Kaohsiung, Taiwan). Purified Water (>18 MΩcm) was used in this study.

### 2.2. Fabrication of Anti-Glare Sol-Gel 

The anti-glare sol-gel formulation was as described by Huang et al. [21] and modified as follows: the desired amounts of TEOS, MTMS, methanol, purified water and nitric acid were added into a 1 L glass container and then magnetically agitated for 24 h at 25 °C. Then, the sol-gel solution was aged at 4 °C for 4 days. The molar ratio of TEOS: MTMS: methanol: HNO_3_: H_2_O was 1: 0.39: 8.39: 0.02: 5.17.

### 2.3. Preparation of Anti-Glare Film Layer

**Figure 2:**
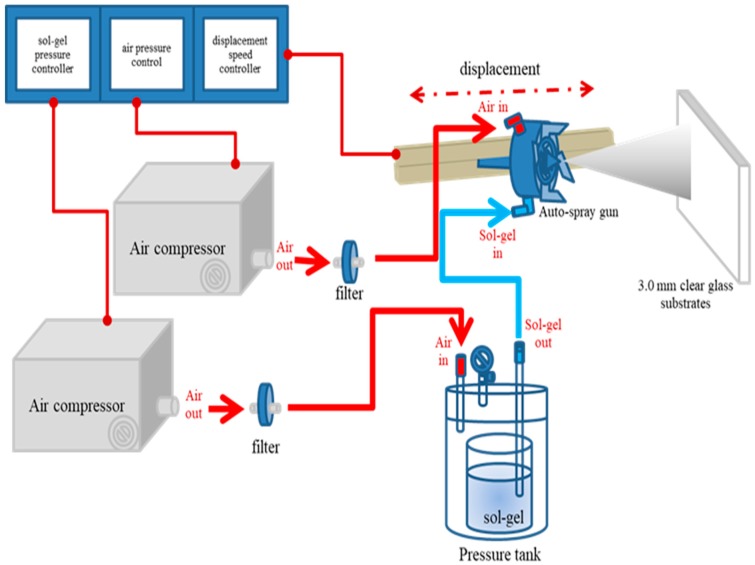
Schematic diagram of the anti-glare thin film production by the auto-spray system.

The glass substrates with diameter of 100 × 100 mm and thickness of 3 mm were ultrasonically cleaned at 50 °C for 30 min and then baked at 80 °C for 1 h. The prepared silica sol-gel samples were deposited onto the cleaned substrates through an automated spray coating system, which was schematically shown in Figure 2. The operating variables of sol-gel deliver pressure, air transport pressure, and the spray gun displacement speed were investigated during the sol-gel deposition under one-pass spray operation conditions. The obtained silica anti-glare film/glass samples were heated at 180 °C for 1 h. In this work, the Auto-spray gun (S-710AD) was purchased from Guan Piin Painting Technology Co., Ltd. (Taiwan). This equipment provides the regulation of sol-gel deliver pressure, air transport pressure, and spray gun displacement speed to spray sol-gel materials on the substrates. Although the distance between the spray gun and substrates can be adjusted, however, for the simplicity of operation, this distance was kept constant in this study. The environmental temperature and humidity were well controlled at 25 °C and 40%, respectively. 

### 2.4. Characterization

The morphologies of the anti-glare thin films were observed using the digit microscope (UPG670, UPMOST technology corp., Taipei, Taiwan). The surface anti-glare property (gloss and haze) of the thin films was measured by the BYK micro-TRI-gloss device (BYK Additives and Instruments Company, Bavarian, Germany), and WGT-S haze meter (Lab-think Instruments Company, Jinan, China). The total transmittance light (TTL) was measured using the HMT MFS-630 angle-adjustable optical measurement analyzer (Hong-Ming Technology Company, New Taipei, Taiwan). Finally, the arithmetical mean deviation of the surface roughness profile (Ra) was measured using the Surface Roughness Tester (TR-200, SAIBORUIXIN, Beijing, China).

### 2.5. Statistical Response Surface Methodology Analysis and Experimental Design 

A classical approach for optimization is to be carried out by changing one variable at a time on an experimental response while keeping other variables unchanged, i.e., one-variable-at-a-time. However, this procedure requires a lot of experimental work. The most serious disadvantage is that it cannot clarify the interactive effect among the independent variables. On the contrary, experimental design is a strategy to conduct the experiments that can change multiple variables at a time and can significantly reduce the test numbers and study time. The experimental design of a simplex with a center point is often used for a linear model [22], while this model cannot present curvature. Three-level factorial, central composite, Box–Behnken, and Doehlert designs can be included in the second or third order models [22,23,24]. The Box–Behnken design includes the midpoint of the edge of the variable space of at least three factors. A central composite design (CCD) is composed of a factor or partial factor design that includes a center point and is augmented with a set of star points that can be used to estimate the bend. The CCD has been considered as one of the most commonly used experimental designs for response surface methodology [22,23,24,25,26,27].

The RSM comprises mathematical and statistical techniques to establish an empirical polynomial equation to correlate the relationship between independent and dependent variables. Following the RSM procedure, the main and interactive effects of the independent variables on the response can be determined according to the coefficients of the corresponding terms in the polynomials. In general, the objective of the RSM is to optimize the process variables to maximize the performance of a system. Bezerra et al. [23] mentioned that the predominant steps for optimization by RSM consist of (1) selection of independent variables, (2) selection of the experimental design method and execution of the experiment, (3) mathematical-statistical processing of experimental data based on polynomial equations, (4) evaluation of the model’s fitness, (5) exploration of the procedure and possibility of optimization, and (6) achievement of the optimum values of the factors. A complete second order polynomial for 3 independent variables is shown in Equation (1):

(1) $$Y=a_{0}+a_{1}X_{1}+a_{2}X_{2}+a_{3}X_{3}+a_{12}X_{1}X_{2}+a_{13}X_{1}X_{3}+a_{23}X_{2}X_{3}+a_{11}X_{1}^{2}+a_{22}X_{2}^{2}+a_{33}X_{3}^{2},$$

where Y represents the dependent variable; X_1_, X_2_ and X_3_ represent the independent variables; a_0_ represents the regression coefficient at the center point; a_1_, a_2_ and a_3_ represent the linear coefficients; a_12_, a_13_ and a_23_ represent the second order interaction coefficients; and a_11_, a_22_ and a_33_ represent the quadratic coefficients. The verified polynomial equation can then be transferred to three-dimensional response surface plots, which may be in favor of indicating the approaching direction of the variables toward the optimal condition. The regression analysis of the relevant experimental data and response plotting were performed using the “Statistica” (StaSoft Inc., Oklahoma, USA) and “Design-Expert 7.0” (STAT-EASE Inc., Minneapolis, USA) statistical suite software. The coefficients of the polynomial equation can be estimated by regressing the experimental data for a specific model after justification by ANOVA. A higher F-value illustrates that more of the variance is likely to be defined by the model and a small one indicates that the variance is mainly attributed to noise. The *p*-value of the ANOVA being less than 0.05 is the basic criterion for judging the significance of the corresponding term in the polynomial equation. The R^2^ value can be used to determine the fitting quality of the proposed model. In addition, the adjusted and predicted R^2^ were also calculated to determine goodness-of-fit of the model without disturbance increased by sample size, and how well a regression model makes predictions, respectively [26,27].

## 3. Results and Discussion

### 3.1. Evaluation of the Most Substantial Factors Affecting Anti-Glare 

The independent variables of sol-gel deliver pressure, air transport pressure, and spray gun displacement speed are supposed to exhibit significant influences on the property of anti-glare. Therefore, these variables were systematically studied using the traditional method by varying one factor at a time and keeping other variables unchanged. The experimental data are summarized in Table 1. 

**Figure 3:**
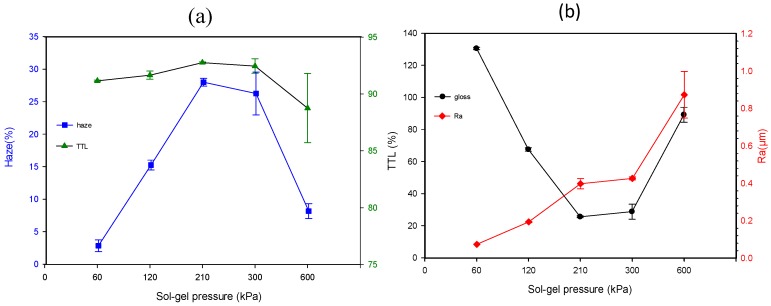
The influence of sol-gel deliver pressure on (**a**) TTL and haze (**b**) gloss and Ra with air transport pressure and displacement speed of 300 kPa and 300 mm/s, respectively.

Figure 3a exhibits the effect of sol-gel deliver pressure on TTL and haze, and Figure 3b displays the trends of gloss and Ra. The TTL slowly increases from 91.2 to 92.8 % as the sol-gel pressure increases from 60 to 210 kPa; meanwhile, a sharp increase of the haze from 2.8 to 28.0% is observed. Both the TTL and haze reach the plateau values as the sol-gel deliver pressure increases to 210 kPa, and then they decrease with further increasing in sol-gel deliver pressure. In contrast, the gloss initially decreases with increasing of sol-gel pressure until 210 kPa; after that, it increases as the sol-gel deliver pressure further increases. On the other hand, the Ra value increases with increasing sol-gel pressure ranging from 60 to 600 kPa. Increasing sol-gel pressure implies the increment of the deposited thickness of the thin film, which is beneficial to reducing gloss and boost haze and TTL. 

**Figure 4:**
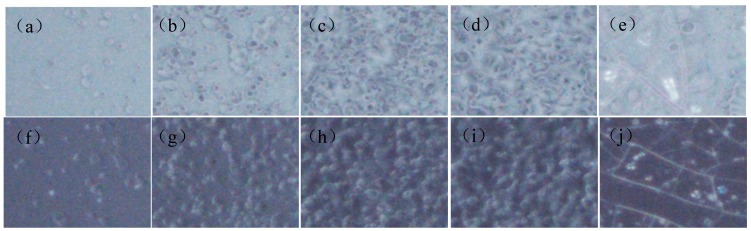
The digital microscope pictures of microstructure morphology of anti-glare samples by different sol-gel deliver pressure: (**a**) 60 kPa (90°); (**b**) 120 kPa (90°); (**c**) 210 kPa (90°); (**d**) 300 kPa (90°); (**e**) 600 kPa (90°); (**f**) 60 kPa (25°); (**g**) 120 kPa (25°); (**h**) 210 kPa (25°); (**i**) 300 kPa (25°); and (**j**) 600 kPa (25°) with air transport pressure and displacement speed of 300 kPa and 300 mm/s, respectively.

The microstructure of anti-glare film is characterized by digital microscope and is shown in Figure 4. It can be seen from the micrograph of 185 times magnification (90° and 25°) that increasing sol-gel pressure results in a coarser surface and larger particle size of the anti-glare thin film, which in turn enhances its scattering effect. In addition, based on the previous research, the deposited sol-gel particles can form a low-refractive SiO_2_ thin film layer, improving the transmittance when the film thickness increases [21].

**Figure 5:**
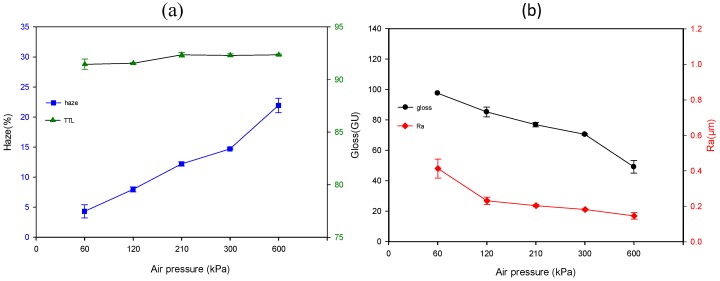
The influence of air transport pressure on (**a**) haze and TTL and (**b**) gloss and Ra with the sol-gel deliver pressure and displacement speed of 120 kPa and 300 mm/s, respectively.

**Figure 6:**
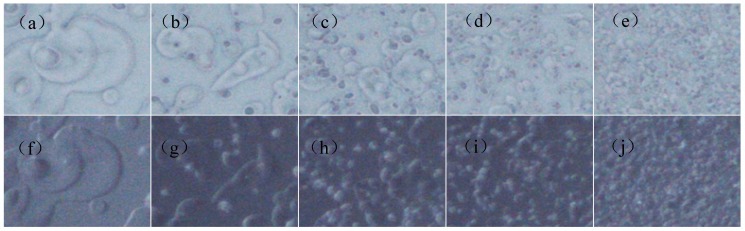
The digital microscope pictures of microstructure morphology of anti-glare samples by different air transport pressure (**a**) 60 kPa (90°); (**b**) 120 kPa (90°); (**c**) 210 kPa (90°); (**d**) 300 kPa (90°); (**e**) 600 kPa (90°); (**f**) 60 kPa (25°); (**g**) 120 kPa (25°); (**h**) 210 kPa (25°); (**i**) 300 kPa (25°); and (**j**) 600 kPa (25°) with the sol-gel deliver pressure and displacement speed of 120 kPa and 300 mm/s, respectively.

However, excess sol-gel pressure may cause the film thickness exceeding the optimum one and generate a thick, smooth, and transparent structure, hindering the anti-glare efficiency. Furthermore, the thin film layers may grow too thick and cause the formation of cracking (Figure 4e,j); as a result, the gloss value will increase, and both the haze and transmittance lower accordingly. Figure 5a,b show the effects of air transport pressure on gloss, haze, TTL, and Ra with the sol-gel deliver pressure and displacement speed are kept at 120 kPa and 300 mm/s, respectively. The results indicate that increasing air transport pressure causes decrement of gloss and Ra, and increment of haze and TTL. The microstructure of the anti-glare film is studied by digital microscope and is shown in Figure 6. Increasing the air transport pressure can produce smaller particles to construct the anti-glare films due to the intensification of atomization.

**Figure 7:**
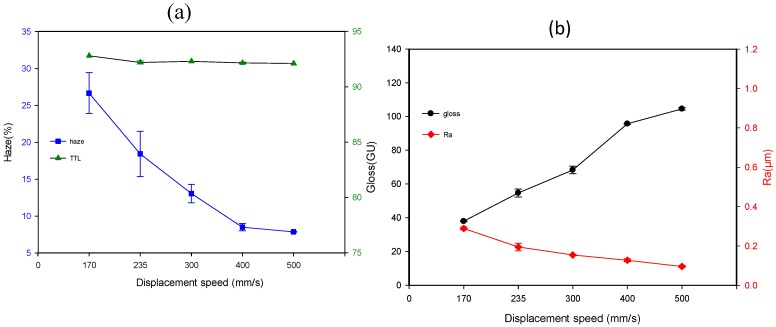
The influences of spray displacement speed on (**a**) haze and TTL, and (**b**) gloss and Ra with the sol-gel deliver and air transport pressures of 120 kPa and 300 kPa, respectively.

These tiny particles are favorable of generating anti-glare property and reducing surface roughness. Figure 7a,b demonstrate the effect of the spray gun displacement speed on gloss, haze, TTL, and Ra while the sol-gel and air transport pressures are kept at 120 kPa and 300 kPa, respectively. The experimental results exhibit that increasing spray gun displacement speed results in a significant increment of gloss and decrease of haze, while the Ra and TTL lower insignificantly with the increase of displacement speed within 170 and 500 mm/s. 

**Figure 8:**
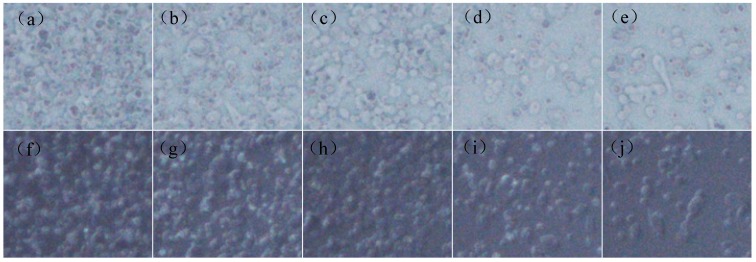
The digital microscope pictures of microstructure morphology of anti-glare sample by different the spray gun displacement speed (**a**) 170 mm/s (90°); (**b**) 235 mm/s (90°); (**c**) 300 mm/s (90°); (**d**) 400 mm/s (90°); (**e**) 500 mm/s (90°); (**f**) 170 mm/s (25°); (**g**) 235 mm/s (25°); (**h**) 300 mm/s (25°); (**i**) 400 mm/s (25°); and (**j**) 500 mm/s (25°) with the sol-gel deliver and air transport pressures of 120 kPa and 300 kPa, respectively.

Figure 8 explores the microstructures of the anti-glare thin films varied with the displacement speeds. It can be seen from the micrograph of 185 times magnification (90° and 25°) that many voids occur and the particle distribution is bumpy. Therefore, the development of anti-glare property with increasing of displacement speed is irrelevant.

Accordingly, the aforementioned single-variable study clearly confirms that these three operating parameters will exert a significant effect on the anti-glare property of the thin film. Therefore, a statistically experimental design based on the CCD and RSM will be conducted to find out the optimum operating condition for maximizing anti-glare.

### 3.2. Statistical Analysis

Although all the responses of gloss, haze, TTL, and Ra are present in Section 3.1; however, at present, gloss and haze are the predominantly evaluated items for anti-glare in industry. Hence, we attempt to optimize the response gloss (Y_1_) and haze (Y_2_) of the anti-glare thin films from the independent variables of the sol-gel deliver pressure (X_1_), air transport pressure (X_2_), and spray gun displacement speed (X_3_). 

Due to the occurrence of critical values of gloss and haze with the factor of sol-gel deliver pressure as shown in Figure 3, a two-level experimental design is not suitable in this system. Hence, a CCD is applied to collect the experimental data in terms of the possible application of a quadratic or a cubic model in this work. Each factor is examined at five levels, coded −1.68, −1, 0, 1 and 1.68. The experimental domains and the levels of the variables investigated are shown in Table 2. The coded values are obtained according to equation (2), where $\chi_{i^{\prime }}$, $\chi_{i}$, and $\Delta \chi_{i}$ represent coded value, real value, and step change of the independent variable i, respectively:

(2) $$\chi_{i^{\prime }}=\frac{\left(\chi_{i}-\chi_{0}\right)}{\Delta \chi_{i}}$$

According to the three-factor CCD, a 16-treatment combination with two repetitive runs at the central point for estimation of the pure error are employed. Table 3 summarizes the experimental results of this CCD. Following the multi-regression technique, ANOVA, and other statistical tests, the proper mathematical mode can be established to disclose the interaction effects between the operation factors, and predict the optimum responses and their corresponding independent variables.

#### 3.2.1. Results of Gloss (Y_1_) Analysis

The sequential model sum of squares (SMSS) and degree of freedom of the linear, quadratic, 2FI (2-factor interaction), and cubic models are shown in Table 4. The linear model includes SMSS from sol-gel deliver pressure (X_1_), air transport pressure (X_2_), and displacement speed (X_3_). The 2FI model is made up of the X_1_X_2_, X_1_X_3_, and X_2_X_3_, while the items of X_1_^2^, X_2_^2^, and X_3_^2^ constitute the quadratic model. The statistical results of Table 4 indicate that incorporating the cubic term cause the model to be aliased. Hence, the full second-order polynomial is established to regress the experimental data. In general, the lower the gloss, the better the anti-glare. Hence, we aim to obtain the experimental conditions of X_1_, X_2_, and X_3_ to reveal minimal gloss value. If the coefficient of an item in the polynomial equation is negative, it means a positive contribution to the anti-glare property. Following the regression techniques, the regression model is expressed in Equation (3). The ANOVA of Equation (3) is summarized in Table 5. The F-value of the model reaches 33.8 with a *p*-value of 0.0002, indicating the significance of this regressed model. To check the validity of the regression model, the F-value of the lack of fit is calculated as 52.5 with a *p*-value larger than 0.05, indicating that the lack of fit is insignificant relative to pure error. The R^2^ is calculated as 0.98, and very close to 1 to justify the adequacy of the regressed model and indicates the experimental data are reasonably consistent with the regressed results. In addition, the predicted R^2^ of 0.84 is in reasonable agreement with the adjusted R^2^ of 0.95. These results also support the adequacy of the regression model. Table 5 also presents the estimates of coefficients of the polynomial equation, standard errors, and 95% confidence interval (CI) of low and high values.

Y_1_ = 25.611 + 9.075X_1_ − 12.497X_2_ + 6.648X_3_ − 7.913X_1_X_2_ + 2.213X_1_X_3_ + 1.688 X_2_X_3_ + 8.338X_1_^2^ + 7.913X_2_^2^ + 1.602X_3_^2^. (3)

The ANOVA exhibits that the three linear terms of X_1_, X_2_, and X_3_ display significant impact on the gloss because their *p*-values are all less than 0.05, among which the most significant factor is air transport pressure (X_2_) owing to the highest F-value and the lowest *p*-value. 

This is because the atomized particles become smaller and scatter more light to reduce the gloss as the air transport pressure increases. In addition, the regression model also implies that decreasing sol-gel deliver pressure (X_1_) and displacement speed (X_3_) can reduce gloss and improve anti-glare property. Reducing sol-gel deliver pressure leads to a small quantity of sol-gel particles being deposited, so that the displacement speed should be slowed down to increase the coverage and uniform distribution of the atomized particles.

In the quadratic terms, X_1_^2^ and X_2_^2^ also reach significant level (*p*-value < 0.05). With regard to the cross-terms, only the interaction effect of X_1_X_2_ reaches a significant level, which implies that the sol-gel deliver pressure (X_1_) and air transport pressure (X_2_) will affect each other and reduce the gloss value. On the contrary, the remaining cross-terms of X_1_X_3_ and X_2_X_3_ are both below the significant level. The significance of X_1_X_2_ can be realized as follows: if the quantity of the sol-gel deliver pressure is large and the air transport is insufficient, then the sol-gel particles cannot be effectively atomized, causing the formation of a thick transparent film. On the other hand, when the sol-gel deliver pressure is small and the air transport pressure is large, finer atomized particles can be expected, but they cannot effectively cover and uniformly distribute on the glass substrate. According to the ANOVA, the *p*-values of the X_1_X_3_, X_2_X_3_, and X_3_^2^ are larger than 0.1, indicating the insignificance of these terms. Therefore, these terms can be omitted from Equation (3), and the regression equation can be simplified to Equation (4):

Y_1_ = 28.131 + 9.075X_1_ − 12.497X_2_ + 6.648X_3_ − 7.913X_1_X_2_ + 7.663X_1_^2^ + 7.239X_2_^2^. (4)

**Figure 9:**
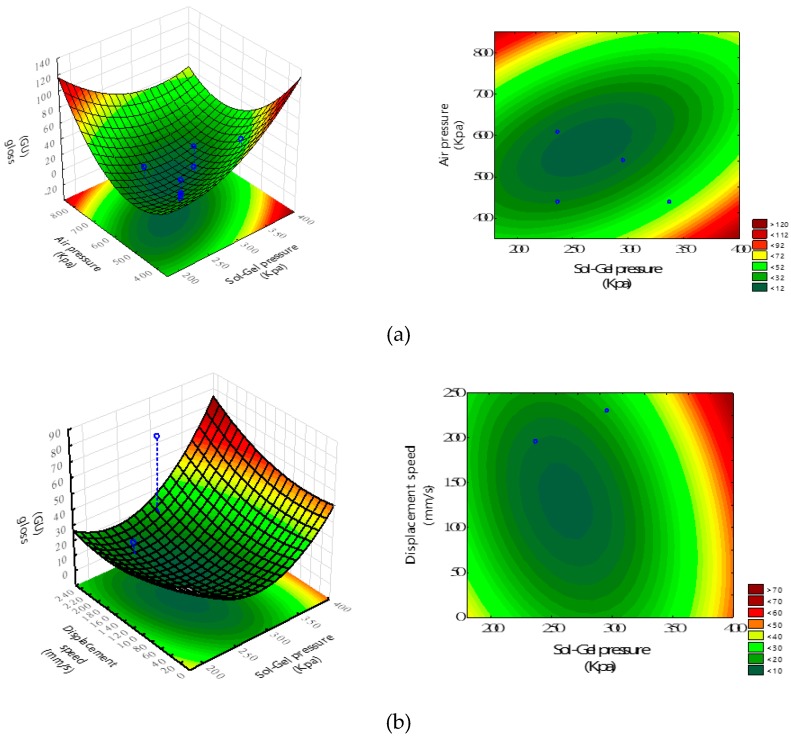

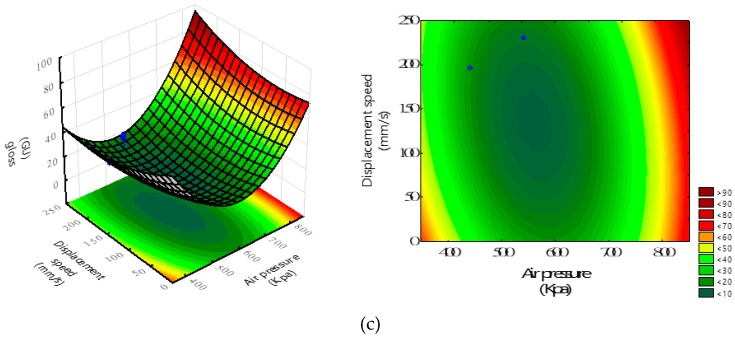
The response surface plots of gloss against the factors of (**a**) sol-gel deliver pressure (X_1_) and air transport pressure (X_2_) with X_3_ of 140 mm/s; (**b**) displacement speed (X_3_) and sol-gel deliver pressure (X_1_) with X_2_ of 560 kPa; and (**c**) displacement speed (X_3_) and air transport pressure (X_2_) with X_1_ of 250 kPa.

This second order regression equation is used to quantitatively describe the relationship between the gloss value and spray-operating parameters. The response surface and contour maps are depicted in Figure 9. According to Equation (4), the optimal (X_1_, X_2_, X_3_) and gloss value are predicted as (250 kPa, 560 kPa, 140 mm/s) and 9.2 GU, respectively. Furthermore, Figure 9 intuitively shows the effect of sol-gel deliver pressure, air transport pressure, and spray gun displacement speed on the gloss of the thin films.

To verify the adequacy of the mathematical model, the experimental runs at the optimal point were carried out. The averaged response gloss value is 9.3 GU, with a negligible error of 1.1% when compared with the predicted one as shown in Table 6. 

#### 3.2.2. Results of Haze (Y_2_) Analysis

In general, a higher haze implies a better anti-glare peculiarity. Therefore, if the coefficient of each independent variable of the regressive model is positive, it means that it can positively contribute to anti-glare effect. The analysis of SMSS for haze is listed in Table 7. The quadratic model is suggested to be adopted to regress the experimental data because the cubic terms cause the model to be aliased. Following the regression procedure, the regression equation for haze is expressed as Equation (5) using the coded values. Table 8 summarizes the results of ANOVA of the regressed model for haze:

Y_2_ = 31.436 − 2.736X_1_ + 5.993X_2_ − 5.069X_3_ + 2.775X_1_X_2_ − 1.175X_1_X_3_ − 1.275X_2_X_3_ − 2.443X_1_^2^ − 3.857X_2_^2^ − 0.675X_3_^2^. (5)

The F-value of the model is 17.8 with a corresponding *p*-value of 0.0011, indicating the significance of this model. The R_2_, R^2^_adj_, and R^2^_pred_ are 0.964, 0.910, and 0.721. The predicted R^2^ is in reasonable agreement with the adjusted R^2^. These results also support the adequacy of the model. In addition, the F-value of the lack of fit is 17.666 (*p* > 0.05), which indicates that it is not significant and shows the adequacy of the model. From Table 8, the multiple regression and the analysis of ANOVA, it can be seen that the sol-gel deliver pressure (X_1_), air transport pressure (X_2_) and displacement speed (X_3_) all reach significant level (*p*-value < 0.05) in the linear terms, among which the most significant variable is air transport pressure (X_2_).

The model also reflects that a slight increase in X_2_ and a slight decrease in X_3_ can produce a more remarkable increment in haze than that by a slight reduce in X_1_ starting from the center point (0,0,0). Under a slow displacement speed, appropriate sol-gel deliver pressure and air transport pressure can effectively atomize the sol-gel particles, and then enhance the surface coverage and uniform distribution of atomized particles on the substrate surface. For the quadratic terms, X_1_ and X_2_ also reached a significant level, which means that these two variables have quadratic effects on haze. Because the coefficients of X_1_^2^ and X_2_^2^ are negative, the extreme values of the haze with X_1_ and X_2_ are supposed to occur. With regard to the cross-terms, only the interaction effect between X_1_ and X_2_ reaches a significant level and exhibits a positive coefficient in the regression model for haze, which implies that the X_1_X_2_ cross-term will affect each other and increase the haze value. The remaining cross-terms of X_1_X_3_ and X_2_X_3_ are both below the significant level. Accordingly, the terms of X_1_X_3_, X_2_X_3_, and X_3_^2^ can be omitted from Equation (5) and the regression equation can be simplified to Equation (6). 

**Figure 10:**
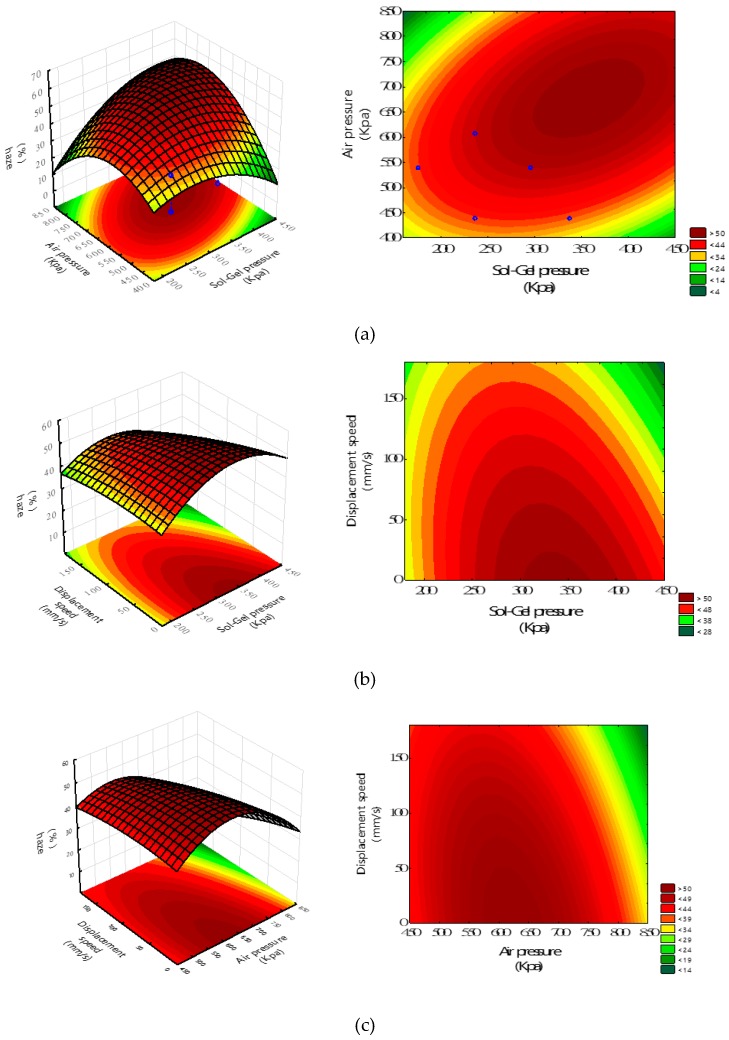
The response surface plots of haze against the factors of (**a**) sol-gel deliver pressure (X_1_) and air transport pressure (X_2_) with X_3_ of 20 mm/s; (**b**) displacement speed (X_3_) and sol-gel deliver pressure (X_1_) with X_2_ of 620 kPa; and (**c**) displacement speed (X_3_) and air transport pressure (X_2_) with X_1_ of 340 kPa.

The response surface and contour maps are depicted in Figure 10. Intuitively, Figure 10 shows the effect of sol-gel deliver pressure, air transport pressure, and spray gun displacement speed on the hazes of the anti-glare glass samples. According to Figure 10, the optimal haze is found as 57.0% with X_1_, X_2_, X_3_ of 340 kPa, 620 kPa, the predicted ability of the mathematical model, we have conducted the experiments and 20 mm/s, respectively, by following the optimized procedure. To examine around the optimal point of X_1_, X_2_, X_3_ of 340 kPa, 620 kPa, and 20 mm/s, respectively, the averaged response haze value is 57.7%, which is very close to the theoretically predicted one of 57.0%, with an error of 1.2%. This result confirms the adequacy of the mathematical model. The experimental results are shown in Table 6.

Y_2_ = 30.374 − 2.736X_1_ + 5.993X_2_ − 5.069X_3_ + 2.775X_1_X_2_ − 2.159X_1_^2^ − 3.573X_2_^2^. (6)

## 4. Conclusions

Following the central composite design and response surface methodology technique, we have successfully reduced gloss by 63.7% and increased haze by 106.1% when compared with the traditionally one-factor-at-a-time method, thus greatly improving the anti-glare performance of the spray-coated thin films in this work. The factors of sol-gel deliver (X_1_) and air transport (X_2_) pressure, and displacement speed of spray gun (X_3_) are proved to significantly affect the formation of anti-glare thin films. Increasing sol-gel delivery and air transport pressures, and reducing the displacement speed, are favorable for reducing the gloss and increasing the haze and transmittance, and then enhancing the anti-glare property. The obtained quadratic polynomial equations can reasonably explain the relationships between the dependent variables (gloss and haze) and the independent variables (X_1_, X_2_, and X_3_). The ANOVA analysis indicates that both the gloss and haze are significantly affected by the factors of X_1_, X_2_, X_3_, X_1_X_2_, X_1_^2^, and X_2_^2^. The main effects of X_1_ and X_2_ on anti-glare property are superior to that of X_3_. The RSM reveals that the optimal points (X_1_, X_2_, X_3_) are around (250 kPa, 560 kPa, 140 mm/s) and (340 kPa, 620 kPa, 20 mm/s) to give the lowest gloss 9.2 GU and the highest haze 57.0%, respectively. These theoretically predicted results are also verified by the real experimental data, confirming the adequacy of the mathematical models.

## Figures and Tables

**Table 1:** Effects of the sol-gel deliver pressure on the gloss, haze, Ra, and TTL of the anti-glare thin films with air transport pressure and displacement speed of 300 kPa and 300 mm/s, respectively.

No.	Sol-Gel Pressure (kPa)	Air Pressure (kPa)	Displacement Speed (mm/s)	Gloss (GU)	Haze (%)	Ra (μm)	TTL (%)
1	60	300	300	130.5	2.8	0.074	91.2
2	120	300	300	67.6	15.3	0.194	91.7
3	210	300	300	25.6	28.0	0.398	92.8
4	300	300	300	28.8	26.3	0.427	92.5
5	600	300	300	89.2	8.2	0.873	88.8

**Table 2:** Experimental domains and coded levels of the independent variables.

Independent Variable	Symbol	Code Level				
		−1.68	−1	0	+1	+1.68
sol-gel deliver pressure (kPa)	X_1_	135.2	176	236	296	336.8
air transport pressure (kPa)(kPa)	X_2_	272	340	440	540	608
displacement speed (mm/s)(mm/s)	X_3_	196	230	280	330	364

**Table 3:** Experimental results of the central composite design.

No.	Sol-Gel Pressure (kPa)	Air Pressure (kPa)	Displacement Speed (mm/s)	Gloss (GU)	Haze (%)
1	176	340	230	32.8	27.3
2	176	340	330	42.0	22.3
3	176	540	230	23.5	35.6
4	176	540	330	31.0	28.6
5	296	340	230	70.2	15.1
6	296	340	330	79.8	8.5
7	296	540	230	20.8	37.6
8	296	540	330	45.6	22.8
9	135.2	440	280	38.6	26.4
10	336.8	440	280	60.5	21.9
11	236	272	280	68.2	11.1
12	236	608	280	28.5	29.2
13	236	440	196	18.7	39.8
14	236	440	364	42.3	18.5
15(C)	236	440	280	25.1	31.0
16(C)	236	440	280	26.0	32..0

**Table 4:** Sequential model sum of squares for gloss.

Sources	Sum of Squares	Degree of Freedom	Mean Square	F-Value	*p*-Value
Mean vs total	26,699.6	1	26,699.6		
Linear vs Mean	3861	3	1287	9.31	0.002
2FI vs Linear	562.804	3	187.6	1.54	0.270
Quadratic vs 2FI	989.678	3	329.9	18.53	0.002
Cubic vs Quadratic	105.134	4	26.3	31.23	0.031 (aliased)
Residual	1.68328	2	0.84		
total	32,219.9	16	2013.7		

**Table 5:** Results of the regression model and ANOVA for gloss.

**Parameter**	**Parameter Estimate**	**Standard Error**		**95% CI Low**	**95% CI High**
Intercept	25.611	2.975		18.332	32.890
X_1_	9.075	1.142		6.281	11.868
X_2_	−12.497	1.142		−15.291	−9.703
X_3_	6.648	1.142		3.854	9.442
X_1_X_2_	−7.913	1.492		−11.563	−4.262
X_1_X_3_	2.213	1.492		−1.438	5.863
X_2_X_3_	1.688	1.492		−1.963	5.338
X_1_^2^	8.338	1.386		4.946	11.730
X_2_^2^	7.913	1.386		4.521	11.305
X_3_^2^	1.602	1.386		−1.790	4.994
**Parameter**	**Sum of Squares (SS)**	**Degree of Freedom (df)**	**Mean Square (MS)**	**F Ratio (F-Value)**	**Probability (*p*-Value)**
Model	5413.482	9	601.498	33.787	0.0002 *
X_1_	1124.634	1	1124.634	63.171	0.0002 *
X_2_	2132.796	1	2132.796	119.800	<0.0001 *
X_3_	603.571	1	603.571	33.903	0.0011 *
X_1_X_2_	500.861	1	500.861	28.134	0.0018 *
X_1_X_3_	39.1613	1	39.161	2.200	0.1886
X_2_X_3_	22.7813	1	22.781	1.280	0.3011
X_1_^2^	644.005	1	644.005	36.174	0.0010 *
X_2_^2^	580.132	1	580.132	32.586	0.0013 *
X_3_^2^	23.7883	1	23.788	1.336	0.2917
Lack of fit	106.413	5	21.283	52.452	0.10443
Pure error	0.405	1	0.405		
Total SS	5520.300	15			

R^2^ = 0.981, R^2^_adj_ = 0.952, R^2^_pred_ = 0.843, Adeq Precision = 18.3.

**Table 6:** The measured optimal condition of lowest gloss and highest haze, and the predicted ones from Equations (3) and (5), respectively.

Items	X_1_ (kPa)	X_2_ (Kpa)	X_3_ (mm/s)	Experimental Value		Predicted Value	Error %
Gloss (GU)	250	560	140	Averaged		9.2	1.1
				9.1	9.3		
				9.5			
				9.3			
Haze (%)	340	620	20	Averaged		57.0	1.2
				57.9	57.7		
				57.7			
				57.6			

**Table 7:** Sequential model sum of squares for haze.

Sources	Sum of Squares	Degree of Freedom	Mean Square	F-Value	*p*-Value
Mean vs total	10,388.7	1	10,388.7		
Linear vs Mean	943.6	3	314.5	12.87	0.005
2FI vs Linear	85.7	3	28.6	1.24	0.359
Quadratic vs 2FI	162.9	3	54.3	7.30	0.019
Cubic vs Quadratic	42.7	4	10.7	1.10	0.080 (aliased)
Residual	1.92	2	0.96		
total	11,625.5	16	726.6		

**Table 8:** Results of the regression model and ANOVA for haze.

**Parameter**	**Parameter Estimate**	**Standard Error**		**95% CI Low**	**95% CI High**
Intercept	31.436	1.923		26.730	36.141
X_1_	−2.736	0.738		−4.542	-0.930
X_2_	5.993	0.738		4.187	7.799
X_3_	−5.069	0.738		−6.875	−3.263
X_1_X_2_	2.775	0.964		0.415	5.135
X_1_X_3_	−1.175	0.964		−3.535	1.185
X_2_X_3_	−1.275	0.964		−3.635	1.085
X_1_^2^	−2.443	0.896		−4.636	−0.250
X_2_^2^	−3.857	0.896		−6.050	−1.665
X_3_^2^	−0.675	0.896		−2.868	1.517
**Parameter**	**Sum of Squares (SS)**	**Degree of Freedom (df)**	**Mean Square (MS)**	**F Ratio (F-Value)**	**Probability (*p*-Value)**
Model	1192.124	9	132.458	17.803	0.0011
X_1_	102.247	1	102.247	13.743	0.0100
X_2_	490.439	1	490.439	65.919	0.0002
X_3_	350.865	1	350.865	47.159	0.0005
X_1_X_2_	61.605	1	61.605	8.280	0.0281
X_1_X_3_	11.045	1	11.045	1.485	0.2688
X_2_X_3_	13.005	1	13.005	1.748	0.2343
X_1_^2^	55.299	1	55.299	7.432	0.0344
X_2_^2^	137.845	1	137.845	18.527	0.0051
X_3_^2^	4.226	1	4.226	0.561	0.4796
Lack of fit	44.140	5	8.828	17.656	0.1787
Pure error	0.500	1	0.500		
Total SS	1236.764	15			

R^2^ = 0.964, R^2^_adj_ = 0.910, R^2^_pred_ = 0.721, Adeq Precision = 13.9.
